# Clinically Translatable Cell Tracking and Quantification by MRI in Cartilage Repair Using Superparamagnetic Iron Oxides

**DOI:** 10.1371/journal.pone.0017001

**Published:** 2011-02-23

**Authors:** Gerben M. van Buul, Gyula Kotek, Piotr A. Wielopolski, Eric Farrell, P. Koen Bos, Harrie Weinans, Anja U. Grohnert, Holger Jahr, Jan A. N. Verhaar, Gabriel P. Krestin, Gerjo J. V. M. van Osch, Monique R. Bernsen

**Affiliations:** 1 Department of Radiology, Erasmus MC, Rotterdam, The Netherlands; 2 Department of Orthopaedics, Erasmus MC, Rotterdam, The Netherlands; 3 Department of Otorhinolaryngology, Erasmus MC, Rotterdam, The Netherlands; 4 Department of Biomechanical Engineering, Delft University of Technology, Delft, The Netherlands; 5 Department of Internal Medicine, Erasmus MC, Rotterdam, The Netherlands; University of Frankfurt - University Hospital Frankfurt, Germany

## Abstract

**Background:**

Articular cartilage has very limited intrinsic regenerative capacity, making cell-based therapy a tempting approach for cartilage repair. Cell tracking can be a major step towards unraveling and improving the repair process of these therapies. We studied superparamagnetic iron oxides (SPIO) for labeling human bone marrow-derived mesenchymal stem cells (hBMSCs) regarding effectivity, cell viability, long term metabolic cell activity, chondrogenic differentiation and hBMSC secretion profile. We additionally examined the capacity of synovial cells to endocytose SPIO from dead, labeled cells, together with the use of magnetic resonance imaging (MRI) for intra-articular visualization and quantification of SPIO labeled cells.

**Methodology/Prinicipal Findings:**

Efficacy and various safety aspects of SPIO cell labeling were determined using appropriate assays. Synovial SPIO re-uptake was investigated *in vitro* by co-labeling cells with SPIO and green fluorescent protein (GFP). MRI experiments were performed on a clinical 3.0T MRI scanner. Two cell-based cartilage repair techniques were mimicked for evaluating MRI traceability of labeled cells: intra-articular cell injection and cell implantation in cartilage defects. Cells were applied *ex vivo* or *in vitro* in an intra-articular environment and immediately scanned. SPIO labeling was effective and did not impair any of the studied safety aspects, including hBMSC secretion profile. SPIO from dead, labeled cells could be taken up by synovial cells. Both injected and implanted SPIO-labeled cells could accurately be visualized by MRI in a clinically relevant sized joint model using clinically applied cell doses. Finally, we quantified the amount of labeled cells seeded in cartilage defects using MR-based relaxometry.

**Conclusions:**

SPIO labeling appears to be safe without influencing cell behavior. SPIO labeled cells can be visualized in an intra-articular environment and quantified when seeded in cartilage defects.

## Introduction

Articular cartilage provides low friction and allows for efficient load bearing and distribution in synovial joints. Cartilage is avascular, aneural and lacks lymphatic drainage. Consequently, natural repair mechanisms are underprovided, giving cartilage a very limited intrinsic regenerative capacity [1]. Current cell-based cartilage repair techniques aim at restoration of the functional properties of damaged cartilage. Human bone marrow-derived mesenchymal stem cells (hBMSCs) and chondrocytes are two cell-types currently used for these approaches [2], [3], [4], [5]. hBMSCs can be either implanted in focal defects, or injected intra-articularly to repair more generalized cartilage lesions like osteoarthritis [4], [5]. Chondrocytes are mainly being used for implantation in focal cartilage defects, a procedure known as autologous chondrocyte implantation (ACI) [2], [3]. Despite the improvements made in cell-based cartilage regeneration in the past decades, repaired cartilage does not entirely resemble native cartilage and can not guarantee a permanent solution at this moment [6].

To elucidate the relevant repair mechanisms and for optimization of cell-based therapies, it is necessary to determine the fate of implanted cells. Superparamagnetic iron oxides (SPIOs), such as ferumoxides, have already been used clinically for cell labeling and *in vivo* cell tracking in dermal oncology, neural regeneration and pancreatic islet transplantation [7]. No negative effects of the labeling procedure have been reported in these initial clinical studies. Before implementing this approach to an orthopedic application, any possible negative influence of SPIO labeling on cell behavior must be ruled out. Besides repopulation of damaged tissue through proliferation and differentiation of hBMSCs, secretion of certain trophic factors by transplanted hBMSCs has more recently been suggested as a possible mechanism by which hBMSCs promote tissue regeneration [8]. These bioactive molecules could provide a regenerative microenvironment to initiate a self-regulated regenerative response. To our knowledge, the effects of SPIO labeling on factors secreted by hBMSCs have not been investigated before. Another point of interest is the feasibility of tracking SPIO-labeled cells within the joint in relation to other anatomical structures, using a clinical magnetic resonance imaging (MRI) protocol, in a model of clinically relevant size. For these purposes, it must be checked how long cells retain the SPIO label and it should be validated if generated MRI signals arise from originally labeled cells. Next to cell visualization, quantification of labeled cells implanted in cartilage defects by MRI could provide an exquisite opportunity to longitudinally map the role and contribution of implanted cells to cartilage repair. The aim of this study was to label hBMSCs using SPIOs and evaluate the effects of iron incorporation on cell viability, long term metabolic cell activity, differentiation and secretion profile. Furthermore we examined the SPIO retention of labeled cells and the capacity of synovial cells to endocytose SPIO from dead, labeled cells. Finally, we studied the use of MRI for accurate intra-articular visualization and quantification of SPIO labeled cells.

## Results

### Labeling effectivity and cell behavior

**Figure 1:**
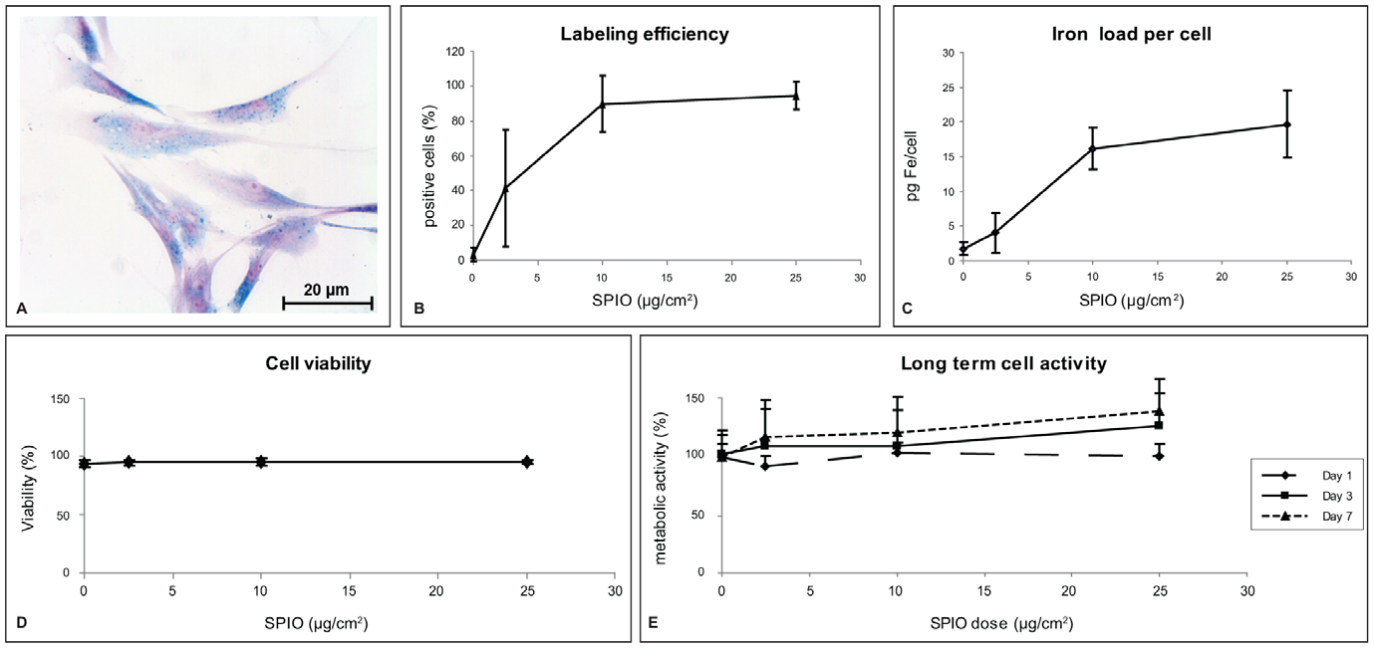
Labeling human Bone Marrow stroma-derived mesenchymal Stem Cells (hBMSCs) using Superparamagnetic Iron Oxides (SPIO). Perl's iron stain showing effective endocytosis of SPIO (A), leading to labeling efficiencies of approximately 95% (B) and a total iron load of approximately 20 pg/cell (C). SPIO labeling did not impair cell viability (D) or subsequent metabolic cell activity (E) at any dose used. Results shown for triplicate samples from three hBMSC donors.

hBMSCs were efficiently labeled with ferumoxides-protamine sulfate complexes (Figure 1A). We observed a labeling efficiency ranging from 41.2±33.5% at an SPIO dose of 2.5 µg/cm2 to 94.5±7.8% at an SPIO dose of 25 µg/cm2 (Figure 1B) with resulting average total iron loads (TILs) of cells ranging from 4.0±2.9 pg/cell to 19.5±6.1 pg/cell (Figure 1C). Both labeling efficiency and TIL increased in relation to SPIO labeling dose up to a dose of 10 µg/cm^2^. The higher SPIO dose of 25 µg/cm^2^ did not significantly increase labeling efficiency (*P = 0.42*) or average TIL (*P = 0.50*). hBMSC viability was not influenced by SPIO labeling for dosages up to 25 µg/cm^2^ (Figure 1D). Additionally, SPIO labeling did not inhibit cell metabolic activity compared to unlabeled controls for at least seven days (Figure 1E). Based on these results an SPIO dose of 10 µg/cm^2^, corresponding to a final labeling concentration of 50 µg/ml SPIO, was considered optimal and used in all further experiments.

**Figure 2:**
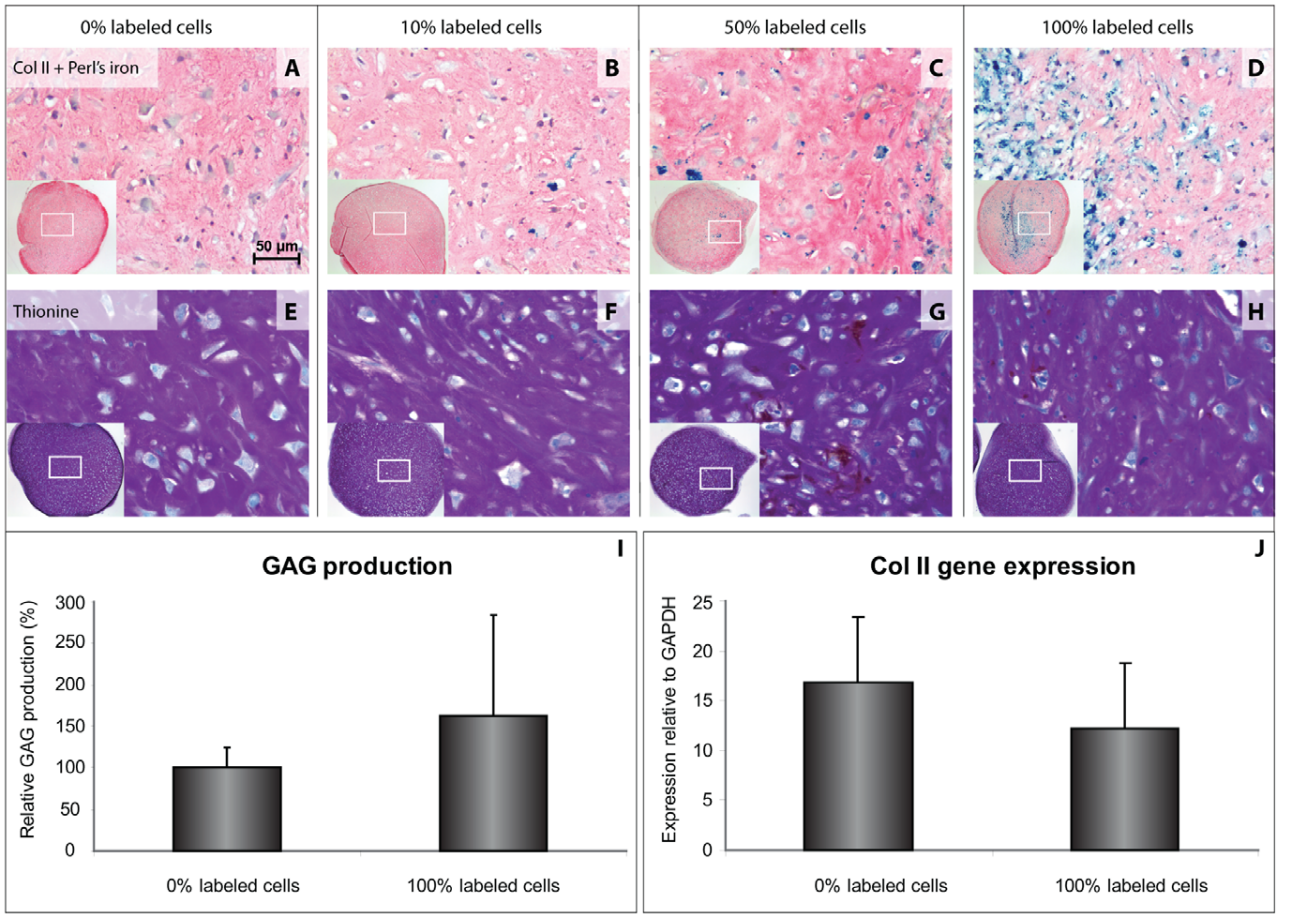
Chondrogenic differentiation of hBMSCs is not affected by SPIO labeling. Collagen II immunohistochemistry and Perl's iron stain demonstrated SPIO labeling to have no effect on pellet size or collagen II production, while iron remained present in differentiated pellets (A–D). Glycosaminoglycan deposition was also unaffected, as can be seen by thionin stain (E–H) and DMB assay (I). Gene expression of collagen II was unaltered by SPIO labeling (J). All analyses were performed after 35 days of differentiation. Insets display low magnification overviews of the entire pellets. Results shown for triplicate samples from two hBMSC donors.

Chondrogenic differentiation was studied in cell pellets consisting of labeled and unlabeled cells at different ratios (0, 10, 50 and 100% of labeled cells). Differentiation was unaffected in all pellets containing labeled hBMSCs (10–100%) compared to control pellets consisting of unlabeled cells only. All pellets displayed a cartilaginous extracellular matrix rich in GAG and collagen type II (Figure 2A–H). Moreover, quantitative measurements revealed SPIO-labeled hBMSCs to produce comparable amounts of GAG (Figure 2I). In addition, gene expression of collagen type II was unaltered in SPIO-labeled hBMSCs after chondrogenic differentiation (Figure 2J).

To evaluate the influence of iron incorporation on cell secretions, we tested six cytokines containing anti- and pro-inflammatory cytokines as well as growth factors. No effect of SPIO-labeling on the secretion profiles was found (Table 1). SPIO-labeled hBMSCs produced comparable quantities of the anti- and pro-inflammatory cytokine IL-6 (*P* = 0.39) and the growth factor VEGF (*P* = 0.31) compared to unlabeled control cells. Furthermore, SPIO labeling did not induce the production of the pro-inflammatory cytokines TNFα and IFNγ, the anti-inflammatory cytokine IL-10 or the growth factor FGF2. These four factors were not detectably secreted by control or SPIO-labeled hBMSCs.

**Table 1:** Cytokine secretion of hBMSCs.

Cytokine (all values in pg/ml)	Control cells	SPIO-labeled cells
**TNFα**	≤d.l.	≤d.l.
**IFNγ**	≤d.l.	≤d.l.
**IL-6**	489±613	330±242
**IL-10**	≤d.l.	≤d.l.
**VEGF**	193±103	265±102
**FGF2**	≤d.l.	≤d.l.

hBMSC secretion profile of four cytokines and two growth factors was not influenced by SPIO labeling. Cytokines were measured in serum free 24 hour supernatant of labeled and unlabeled cells. SPIO did not induce the secretion of pro-inflammatory cytokines (TNFα, IFNγ), nor did it alter the secretion of the anti- and pro-inflammatory cytokine IL-6 or the growth factor VEGF. The anti-inflammatory factor IL-10 and growth factor FGF2 were not secreted at a detectable level by control or SPIO-labeled cells. TNF-α: tumor necrosis factor-alpha, IFN-γ: interferon-gamma, IL: interleukin, VEGF: vascular endothelial growth factor, FGF: fibroblast growth factor, ≤d.l.: below detection level (10 pg/ml). Results shown for duplicate samples from five hBMSC donors.

### Cell visualization by MRI

**Figure 3:**
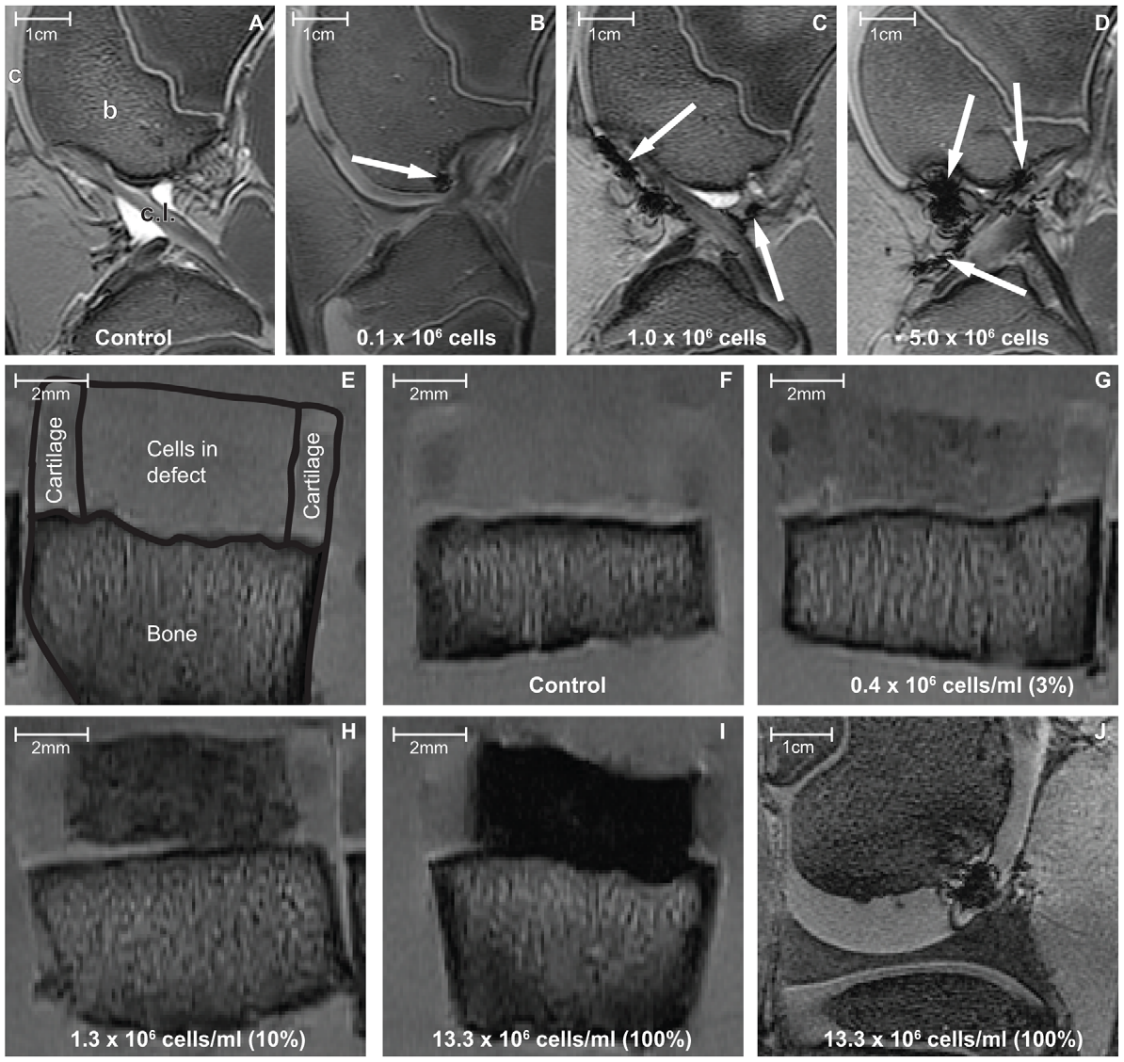
MRI visualization of SPIO-labeled hBMSCs in an intra-articular environment. Injected cells were visualized in a dose dependent manner (A–D, in-plane resolution 273×273 µm; slice thickness 600 µm). Cells were found scattered throughout the joint (arrows) and could be discerned from intra-articular structures. Labeled cells implanted in cartilage defects *in vitro* were homogeneously distributed and imaged in a comparable way; dose dependent and clearly distinguishable from structures like cartilage and bone (E–I; in-plane resolution 78×78 µm; slice thickness 1500 µm). After implanting cells in a cartilage defect in an intact porcine knee, MRI showed the cells inside the defect (J; in-plane resolution 273×273 µm; slice thickness 400 µm). In (A); b: bone; c: cartilage; c.l.: cruciate ligament. Intra-articular cells in porcine knees were imaged as a single sample for one donor. hBMSCs implanted in cartilage defects *in vitro* were imaged in duplicate samples from two hBMSC donors.

All cell doses injected intra-articularly could be visualized by MRI. The number of injected cells was related to the number and extent of areas with signal loss detected by MRI (Figure 3A–D, arrows). Injected hBMSCs were found scattered throughout the joint, clearly distinguishable from anatomical structures in all conditions. In addition, presence of labeled cells did not interfere with regular evaluation of knee anatomy.

For cell implantation experiments we created cartilage defects of 75 µl in porcine osteochondral plugs *in vitro* (Fig. S1A). Labeled hBMSCs implanted in cartilage defects were homogenously distributed and could be clearly distinguished from surrounding bone and cartilage by MRI. The extent of signal loss in the seeded plugs was dependent on the labeled cell dose used (Figure 3E–I). The minimal labeled cell concentration we could visualize was 0.4×10^6^/ml. To show the potential applicability of this technique in a clinical ACI-like procedure, we subsequently implanted labeled hBMSCs in a cartilage defect in an intact porcine knee. In this setting the SPIO-labeled cells were also clearly distinguishable from the surrounding tissues (Figure 3J). Post MRI, Perl's iron stain confirmed SPIO positive cells to be present inside the defect (Fig. S1B).

**Figure 4:**
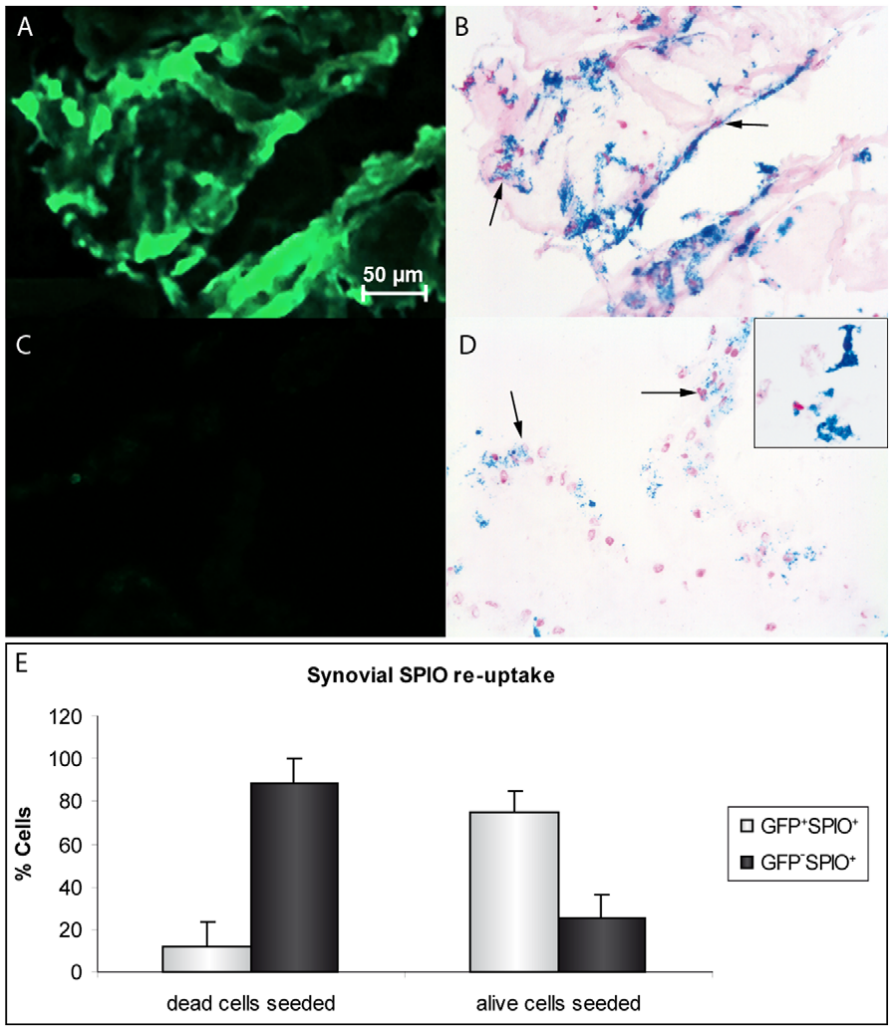
SPIO re-uptake by human synovial explants. Fluorescent images and Perl's iron stain of GFP-SPIO co-labeled chondrocytes seeded on synovial explants. GFP^+^-SPIO^+^ cells, indicating originally seeded chondrocytes, were seen in samples seeded with living cells (A,B). In samples seeded with dead cells, GFP̂-SPIO^+^ cells were found, indicating SPIO re-uptake by synovial cells (C,D). Furthermore, extracellular SPIO aggregates were observed attached to the synovial explants (D, inset). Approximately 90% of SPIO^+^ cells in samples seeded with dead chondrocytes were not originally labeled cells, compared with 25% of SPIO^+^ cells in samples seeded with living cells (E). Arrows indicate SPIO containing cells. Results shown for triplicate samples from two synovium donors.

A point of interest is whether observed areas with signal loss are generated by originally labeled cells, since SPIO remains in the joint after labeled cells die. The joint cavity is lined by an inner layer of synovial tissue that contains macrophages. We investigated the capacity of synovial cells to endocytose SPIO from labeled cells, by seeding dead or living GFP-SPIO co-labeled chondrocytes on synovial tissue explants. In the synovium samples seeded with living GFP-SPIO co-labeled cells, GFP^+^ cells were observed immediately after seeding (Fig. S2A). Moreover, GFP^+^-SPIO^+^ cells were found after the co-culture period of five days, suggesting these cells to be originally labeled chondrocytes (Figure 4A and 4B). When seeded with dead GFP-SPIO co-labeled cells, fluorescence microscopy confirmed the vast majority of cells to have died directly following the freeze-thaw procedure (Fig. S2B). After five days of co-culture, 88.4±11.4% of the SPIO^+^ cells were GFP̂ in these samples, indicative of iron re-uptake by synovial cells (Figure 4C–E). Next to this, extracellular SPIO aggregates were found attaching to the synovial membrane (Figure 4D inset). In samples seeded with living chondrocytes, 25.5±10.5% of SPIO positive cells showed no GFP signal either. Whether this was due to extinguishing GFP signal from originally labeled chondrocytes, synovial re-uptake of SPIO from living cells, or caused by iron re-uptake from seeded cells that died during the co-culture period, could not be determined.

### Cell quantification by MR-based relaxometry

**Figure 5:**
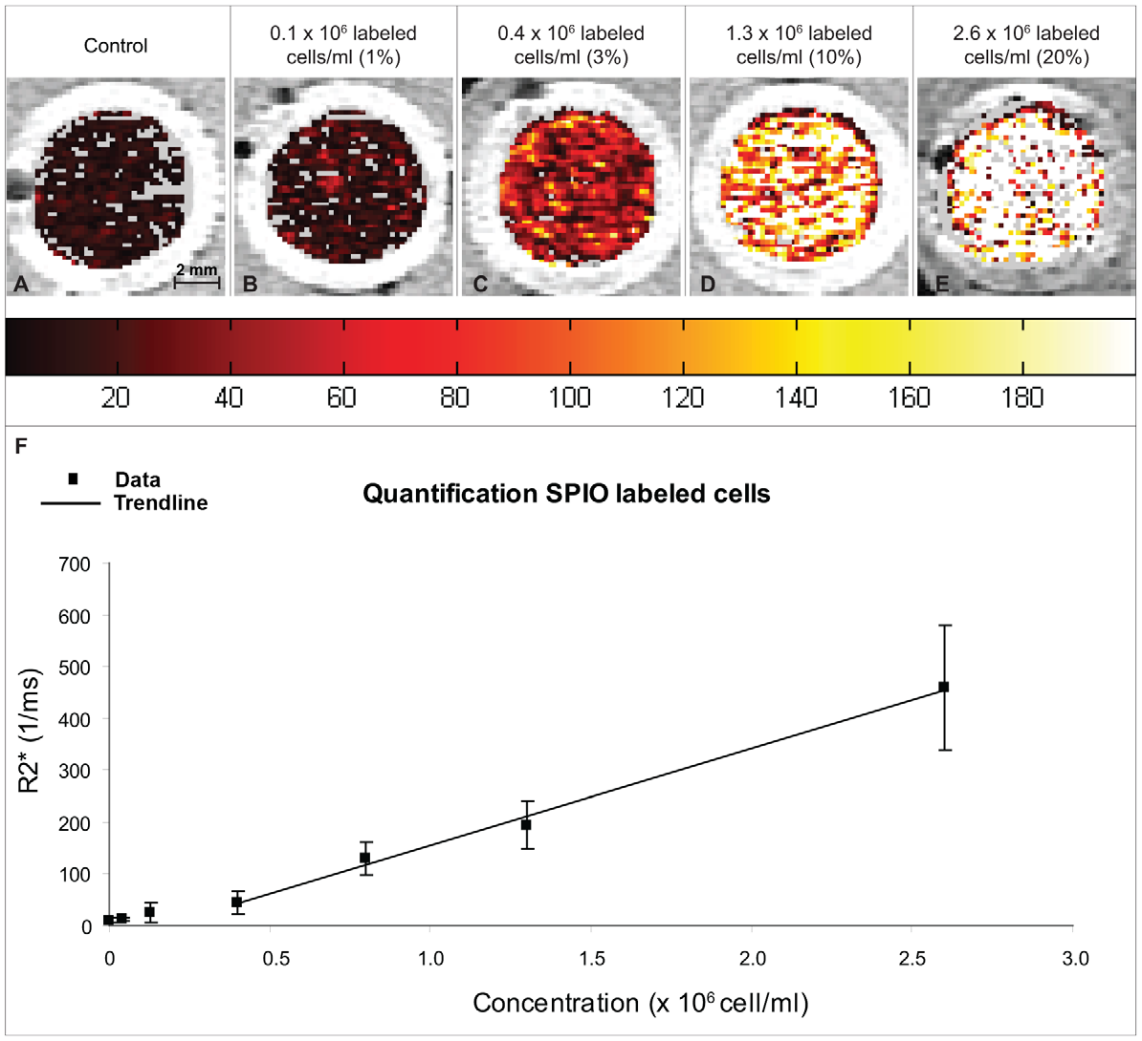
Quantification of SPIO-labeled cells implanted in cartilage defects *in vitro*. Transverse R2* maps from identical osteochondral plugs as shown in figure 3, visualize the differences in labeled cell concentrations (A–E; in-plane resolution 156×156 µm, slice thickness 700 µm). A linear relationship between labeled cell concentration and R2* value was found in a range of 0.4×10^6^–2.6×10^6^ cells/ml (F). Grey voxels inside the defects represent positions where R2* values could not be obtained due to a too low (mainly in A,B) or too high (mainly in E) local concentration of SPIO. Results shown for duplicate samples from two hBMSC donor for samples containing 0; 1.3×10^5^; 4.0×10^5^ and 1.3×10^6^ cells/ml. Samples containing 4.0×10^4^; 8.0×10^5^ and 2.6×10^6^ cells per ml were scanned in duplicates for one hBMSC donor.

In addition to visualization of seeded cells by MRI, we were able to quantify the concentration of hBMSCs seeded in defects *in vitro* by applying a R2* mapping technique, in which R2* = 1/T2* (Figure 5A–E). A linear relationship between R2* values and labeled cell concentrations of 0.4×10^6^–2.6×10^6^ cells/ml was found (Figure 5F). R2* values ranged from 45±22 - 460±120 ms^−1^ for these labeled cell concentrations. Below 0.4×10^6^ cells/ml, SPIO-generated R2* signal was insufficient for reliable differentiation from defects seeded with unlabeled hBMSCs. At the very high labeled cell concentration of 13×10^6^ labeled cells/ml R2* values could no longer be determined, since the values exceeded those measurable with our clinical acquisition parameter range. At this high SPIO concentration a signal-to-noise ratio of approximately 1.6 was obtained, which signifies that the signal loss was not reliably discernable from background noise.

## Discussion

Cell-based cartilage repair techniques are developing rapidly, and the use of hBMSCs as a treatment for focal cartilage defects and osteoarthritis shows promising results [5], [9]. Clinically translatable cell tracking represents a vital tool to aid in the elucidation of the repair mechanisms of these therapies, and in further improving them. In this study we have shown that SPIO labeling of hBMSCs is effective and does not impair cell viability, long term metabolic cell activity, chondrogenic differentiation, or the secretion of a panel of six cytokines involved in inflammation and tissue regeneration. We were able to accurately visualize intra-articular SPIO-labeled hBMSCs and quantify cells seeded in localized cartilage defects by means of an R2* mapping MRI technique.

Regarding the influence of SPIO labeling on chondrogenic capacity of BMSCs, some discrepancies can be found in recent reports. Some groups mention no influence of SPIO labeling on chondrogenic differentiation of BMSCs [10], [11], whereas others do report a negative effect [12], [13]. These conflicting data could be due to the wide variety in protocols for SPIO labeling and chondrogenic differentiation used. Furthermore, the influence of cellular iron load on chondrogenic differentiation has been described [13], [14]. As reported in this paper we found no SPIO-related negative effects in our chondrogenic assays with freshly isolated cells. However, we have observed inhibitory effects if we used cryopreserved cells in pellets containing ≥50% labeled cells, although pellets with 10% labeled cells appeared unaltered (Fig. S3A–D). Therefore we recommend further investigation regarding this subject when using cryopreserved hBMSCs.

Besides cell proliferation and differentiation, other hBMSC capacities are relevant in regenerative medicine as well. Recently, secretion of trophic factors was postulated as a possible role for hBMSCs in cartilage regeneration [8]. We demonstrated that the hBMSC secretion profile of six important factors involved in inflammation and cell growth was not affected by SPIO. Previous research has shown that SPIO labeling does not impair hBMSC immunomodulatory capacities in a mixed lymphocyte reaction [15]. Furthermore, it has been described that gene expression of neural stem cells, with the exception of iron homeostasis related genes, remains largely unaltered after SPIO labeling [16]. We report, to our knowledge, for the first time the influence of SPIO labeling on cell secretions at a protein level. These findings could be relevant for other regenerative medicine fields using hBMSCs, like cardiology, neurology and organ transplantation [17]. In summary we found no deleterious effects of our labeling procedure on cell functionality. When labeling only part of the applied cells, which in our experiments sufficed for accurate intra-articular MRI traceability, the risk of possible negative effects will even be further diminished.

In order to translate our labeling method to a cell tracking approach in an intra-articular environment, we investigated the MRI traceability of clinically used amounts of SPIO-labeled cells in a joint model of a clinically relevant size. Jing et al. previously visualized cells labeled with SPIO in rabbit knee joints by MRI [18]. Through the use of a clinical phased-array head coil and specific MRI parameters we were able to increase MRI resolution (in plane resolution 273×273 µm), which is higher than in other studies performing intra-articular cell tracking or general clinical anatomical intra-articular imaging by MRI [19], [20]. This enabled dose-dependent visualization of labeled cells and discrimination from intra-articular structures like cruciate ligaments, cartilage and subchondral bone. Following our *in vitro* cell implantation experiments, we visualized SPIO-labeled cells implanted in an ACI-like procedure in an intact porcine knee. Air artifacts show up as hypointensities on MRI, which interferes with the signal loss generated by labeled cells. To avoid these artifacts, we were obliged to perform this operation under water. This is impracticable in a clinical setting indicating that immediately post-operative this technique can be used to visualize cells that remained in the defect, but that the technique is unsuitable to discern labeled cells that might have leaked in the intra-articular space. These leaked-out cells could be identified after intra-articular air has resolved, which in our personal clinical experience occurs within a day.

SPIO for cell-labeling has great clinical potential, but unfortunately the label will dilute upon cell division. This could limit the duration that cells can be accurately detected by MRI. Previous results from our group and others show an approximate 75% decrease in SPIO positive cells after 7 days of expansion in culture [21] and a total loss of iron after 5–8 cell divisions in rapidly dividing HeLa cells [22]. On the other hand, duration of traceability increases with slower proliferating cells, and SPIO could be detected after 44 days in non-dividing human MSCs [22]. Accordingly, our group previously reported traceability in vivo of SPIO labeled human MSCs in a subcutaneous mouse model for at least seven weeks post labeling [10]. In additional experiments we found a marked decrease in SPIO-generated signal voids from proliferating labeled hBMSCs over a period of 14 days in vitro, representing 5–6 cell divisions (data not shown). How long SPIO labeled cells can be detected in an intra-articular environment remains to be studied in an *in vivo* model.

Another point regarding accurate detection of labeled cells is the fact that cell death will not result in the disappearance of the label. SPIOs released from these cells could lead to misinterpretation of MR images. Pawelczyk et al. have previously shown approximately 10–20% of local macrophages to become SPIO positive after injection of SPIO-labeled BMSCs in a subcutaneous inflammation mouse model [15]. Synovium contains the majority of macrophages present in synovial joints. We observed the vast majority of SPIO^+^ cells to be non-originally labeled after applying dead, labeled cells on synovial explants. In the case of cell implantation in a cartilage defect, SPIO re-uptake by host cells might be of less consequence because there is no direct contact between the cells seeded in the defect and the synovial membrane. Nonetheless, before clinical application we recommend *in vivo* animal studies using intra-articular cell tracking by MRI in combination with histological confirmation of the localization of originally labeled cells. This would enable the correct interpretation of obtained MR images.

Besides visualizing cells, we were able to quantify beforehand known cell concentrations implanted in cartilage defects in a clinically relevant cell-concentration range. To our knowledge, this has not been reported before in a model comprising biological tissue. Rad et al. were unsuccessful in translating their SPIO-labeled cell quantification experiments to an *ex vivo* setting [23], and Politi et al. did not validate their *ex vivo* quantification results afterwards [24]. Since the original concentration of applied cells is known in an ACI procedure, and seeded in a confined volume, it is possible to quantify the total amount of labeled cells and follow them in the same patient in subsequent MRI sessions. Since both hBMSCs and chondrocytes can be labeled using SPIO [25], [26], these findings indicate the potential of SPIO labeling for tracking cells in numerous orthopedic treatments and many other regenerative medicine fields [27]. Cell visualization and especially quantification using MRI can be a major step towards improvement in all these applications.

In summary, we consider SPIO labeling to hold great potential for clinically translatable cell tracking using MRI in cell-based cartilage repair. In an experimental setting this would show the distribution and diffusion of the applied cells, thereby elucidating the regenerative mechanisms and providing opportunities to improve current repair strategies. The use of MRI has the advantage of simultaneous cell tracking and evaluation of cartilage regeneration in one MRI session.

## Materials and Methods

### Ethics Statement

All human samples were obtained after approval by the Erasmus MC medical ethical committee. hBMSCs were isolated from heparinized femoral-shaft marrow aspirate of patients undergoing total hip arthroplasty (after written informed consent; protocol # MEC-2004-142). Articular cartilage and synovial explants were obtained as redundant material from patients undergoing total knee replacement surgery. All patients implicitly consented to the use of these tissues for scientific research (protocol # MEC-2004-322).

### Cell isolations and cultures

hBMSCs and human chondrocytes were isolated and cultured using previously described procedures [28], [29]. All isolated cells or explants were cultured in DMEM containing 10% FCS, 50 µg/ml gentamicin and 1.5 µg/ml fungizone (defined as standard medium) unless stated otherwise. hBMSCs were cultured using additional 1 ng/ml FGF2 and 1×10^−4^ M vitamin C. All hBMSCs and chondrocytes were used at passage 2–5 in labeling experiments.

### SPIO labeling

SPIO labeling was performed using ferumoxides (Endorem®, Guerbet S.A., Paris, France) complexed to protamine sulfate (LEO Pharma N.V., Wilrijk, Belgium) as described earlier [25]. For removal of extracellular iron, cells were washed with PBS containing heparin 10 U/ml (LEO Pharma B.V., Breda, the Netherlands). SPIO labeling mix was made at a constant concentration of 100 µg/ml ferumoxides with 5 µg/ml protamine to ensure identical particle formation [30]. Cells were labeled with doses ranging from 2.5–25 µg/cm^2^ of ferumoxides.

### Labeling efficiency, chondrogenic differentiation and functional assays

Histological iron detection was performed using a Perl's iron stain (Klinipath BVBA, Duiven, the Netherlands) according to the manufacturer's protocol. Labeling efficiency was determined in triplicate samples for three donors by manually counting labeled cells stained with Perl's iron [25]. Total iron load was measured in triplicate samples from three donors by inductively coupled plasma - optical emission spectroscopy [25]. Cell viability was evaluated by trypan blue exclusion. After labeling, cells were seeded in 96-well plates at a density of 10,000 cells per well. AlamarBlue® (Invitrogen, Breda, the Netherlands) was used according to manufacturer's protocol for longitudinal measurement of metabolic cell activity at day 0, 3 and 7.

Chondrogenic differentiation was performed in triplicate samples on hBMSCs pellets from two donors according to previously reported protocols [31]. Chondrogenically differentiated hBMSCs were stained using a collagen II monoclonal antibody (II-II6B3; Developmental Studies Hybridoma Bank, Iowa City, IA) and thionine stain [31], [32]. hBMSCs used for gene expression analysis and glycosaminoglycan (GAG) measurements were chondrogenically differentiated in alginate beads [33] using an otherwise identical differentiation protocol. RNA extraction, cDNA synthesis, real-time RT-PCR and collagen II primers for gene expression analysis were described earlier [34]. GAG measurements were performed using a dimethylmethylene blue (DMB) assay [35].

To investigate the influence of SPIO labeling on cytokines secreted by hBMSCs, labeled and unlabeled cells from five donors were cultured for 24 hours in serumfree standard medium supplemented with ITS+1 (serum replacement; BD Bioscience, Bedford, MA) in a 1∶100 v/v ratio. Secretion of tumor necrosis factor-alpha (TNF-α), interferon-gamma (IFN-γ), interleukin (IL)-6, IL-10, vascular endothelial growth factor (VEGF) and fibroblast growth factor 2 (FGF2) were analyzed in duplicate samples using a human Cytokine Bead Array flexsets (BD Bioscience, Breda, the Netherlands) according to the manufacturer's protocol. Flow cytometric measurements were performed on a FACSCanto II (BD, San Jose, CA, USA) and analyzed using FACSDiva software version 6.1.2 (BD Biosciences).

### Intra-articular cell injections and cell implantations

Due to the *ex vivo* nature of our MRI experiments, all cells were fixed in 4% formalin prior to injection or implantation. SPIO-labeled hBMSCs were injected *ex vivo* into porcine knees (Yorkshire × Landrace, 35 kilograms, age 4–5 months) in a dose range of 0.1×10^6^ to 5×10^6^ labeled cells (injected in 100 µl physiological saline). For cell implantation, osteochondral plugs with a diameter of 8 mm were harvested from porcine femoral condyles *ex vivo*. Circular full-cartilage defects of 6 mm diameter (approximate volume of 75 µl) were created in those plugs. Cells from two donors were suspended in 1% Agar (Fluka BioChemika, Sigma-Aldrich, Buchs, Switzerland) in saline before implantation. A total of 1×10^6^ cells were seeded per defect *in vitro* (cell concentration of 13×10^6^ cells/ml), containing 0.3 – 100% labelled cells (i.e. 0.04×10^6^–13×10^6^ labeled cells/ml). Cell implantation was also performed *ex vivo* in an intact porcine knee according to previously described autologous chondrocyte implantation (ACI) procedures [2]. The knee joint was opened and a circular cartilage defect was created (diameter 6 mm; volume 75 µl). A periosteal flap was sutured on top of the defect using PDS 6-0 sutures (Johnson & Johnson, New Brunswick, New Jersey) and fibrin glue (Tissucol® Duo 500, Baxter, Uden, the Netherlands) was applied at 90% of the edges of the flap as a sealant. 1×10^6^ SPIO-labeled cells suspended in 1% Agar, were seeded into the defect and fibrin glue was used to seal the final 10% of the periostal flap margin. The joint was closed in layers afterwards. All surgical procedures - except the cell seeding and fibrin glue application - were performed under water in order to prevent air artifacts on MRI.

### Validation SPIO-generated signal

To validate if SPIO-generated signal was related to originally labeled cells, or that SPIO particles were taken up by host cells, human green fluorescent protein (GFP) transfected chondrocytes were co-labeled with SPIO. Cells were lentivirally transduced and constitutively expressed GFP under the control of a CMV promoter (SHC003 vector, Sigma-Aldrich Mission®, shRNA library). To investigate the fate of SPIO after cell death, we considered the cell type used for labeling less relevant and chose chondrocytes over hBMSCs due to their substantially higher transduction efficiency. 25×10^4^ GFP^+^-SPIO^+^ cells were seeded in duplicates on synovial explants from two human donors. Cells were alive or killed (by repeated free-thaw cycles) before seeding. Cells and synovial explants were co-cultured for five days. Fluorescent images were made from frozen sections using an Axiovert s100 microscope (Carl Zeiss B.V., Sliedrecht, the Netherlands). Subsequently, samples were stained using Perl's iron stain and corresponding fields of view were evaluated by light microscopy. GFP and SPIO containing cells were determined by manually counting a minimum of 100 cells per sample.

### Magnetic Resonance Imaging

Scanning was performed on a clinical 3.0T MRI scanner (General Electric Healthcare, Milwaukee, Wisconsin). A 3D SPGR sequence was used to scan labeled hBMSCs *in vitro* (TR 87.7 ms, TE 15.1 ms, FOV 17 mm, matrix 512×512, scanning time 11 min. 35 sec.) in combination with a custom made receive-only surface coil with a diameter of 1 cm. Scanning of injected or implanted hBMSCs in intact porcine knees was performed using a FIESTA-3D sequence (TR 7.9 ms, TE 2.2 ms, FOV 140 mm, matrix 512×256, scanning time 28 min. 46 sec.) and a clinical phased-array head coil. Images of hBMSCs implanted in defects *in vitro* were obtained by a Spin Echo sequence (TR 1500 ms, TE 40 ms, FOV 40 mm, matrix 512×512, scanning time 14 min. 42 sec.) and a custom made receive-only surface coil with a diameter of 3 cm. R2*-mapping was performed in duplicate samples for two hBMSC donors by a SPGR sequence (TR 45 ms, TE 4–120 ms, FOV 40 mm, matrix 256×256, scanning time 21 min. 21 sec.). R2* values were calculated and validated by mono-exponential curve fitting (MATLAB 7.0.1, MathWorks, MA). Voxels with an r^2^<0.95 were excluded from the measurements.

### Statistical analysis

Statistical differences between different treatment groups were analysed by the paired Student t-test (normality test p>0.05). P-values of ≤0.05 were considered statistically significant. All numerical data are presented as mean ± standard deviation.

## Supporting Information

Figure S1 ***In vitro*** **and** ***ex vivo*** **cell implantation procedures.** Osteochondral plugs with a diameter of 8 mm were created from the femoral part of porcine knees (A). *Ex vivo*, post-MRI Perl's iron stain shows macroscopically the presence of blue, iron containing cells in the defect (B). Dotted line in (A) represents created defect. Intra-articular cell implantation in a porcine knee was performed for one donor.
(TIF)

Figure S2 **Cell seeding on synovial explants.** Fluorescent images directly after seeding GFP^+^-SPIO^+^ cells on synovial explants showing abundant GFP signal in samples seeded with living cells (A), while GFP signal is absent in samples seeded with dead cells (B). This confirmed that the vast majority of killed cells had not survived the multiple freeze-thaw procedures. Representative images are shown for triplicate samples from two synovium donors.
(TIF)

Figure S3 **Chondrogenic differentiation of cryopreserved SPIO-labeled hBMSCs.** Perl's iron stain (A–D) and collagen II immunohistochemistry (A–C) of chondrogenically differentiated cryopreserved hBMSCs. SPIO labeling of 10% of the cells did not influence pellet size or collagen II deposition compared to control cells (A and B). Using 50% of SPIO-labeled cells did negatively influence these outcome measures (C). Pellets consisting of 100% of labeled cells disintegrated within 7 days, showing viable and iron containing cells on a cytospin (D). Results shown for triplicate samples from two hBMSC donors.
(TIF)
