# Supplementary figures and images for: Clinically Translatable Cell Tracking and Quantification by MRI in Cartilage Repair Using Superparamagnetic Iron Oxides
Source: PLoS One. 2011 Feb 23;6(2):e17001. doi: 10.1371/journal.pone.0017001 (PMC3044153; doi:10.1371/journal.pone.0017001)

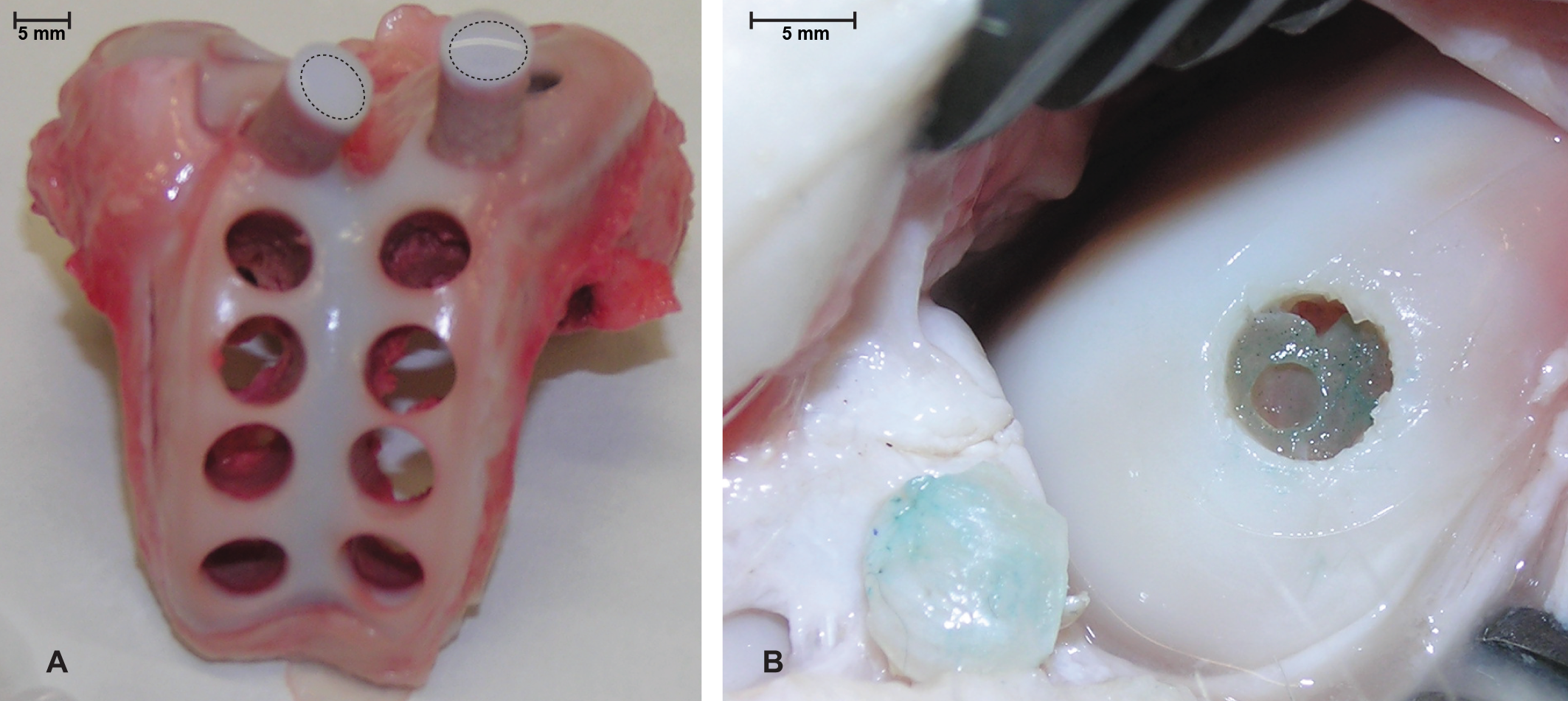

Supplement: Figure S1 — In vitro and ex vivo cell implantation procedures. Osteochondral plugs with a diameter of 8 mm were created from the femoral part of porcine knees (A). Ex vivo, post-MRI Perl's iron stain shows macroscopically the presence of blue, iron containing cells in the defect (B). Dotted line in (A) represents created defect. Intra-articular cell implantation in a porcine knee was performed for one donor. (TIF) [file pone.0017001.s001.tif]

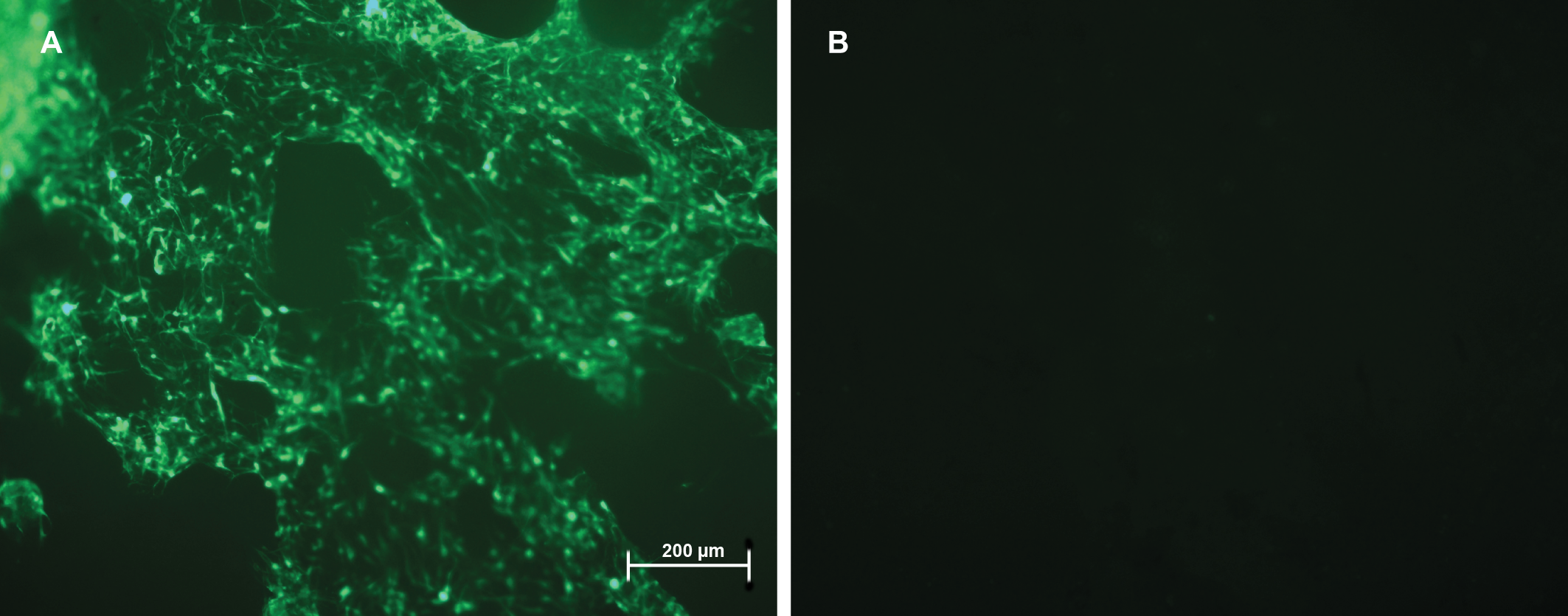

Supplement: Figure S2 — Cell seeding on synovial explants. Fluorescent images directly after seeding GFP+-SPIO+ cells on synovial explants showing abundant GFP signal in samples seeded with living cells (A), while GFP signal is absent in samples seeded with dead cells (B). This confirmed that the vast majority of killed cells had not survived the multiple freeze-thaw procedures. Representative images are shown for triplicate samples from two synovium donors. (TIF) [file pone.0017001.s002.tif]

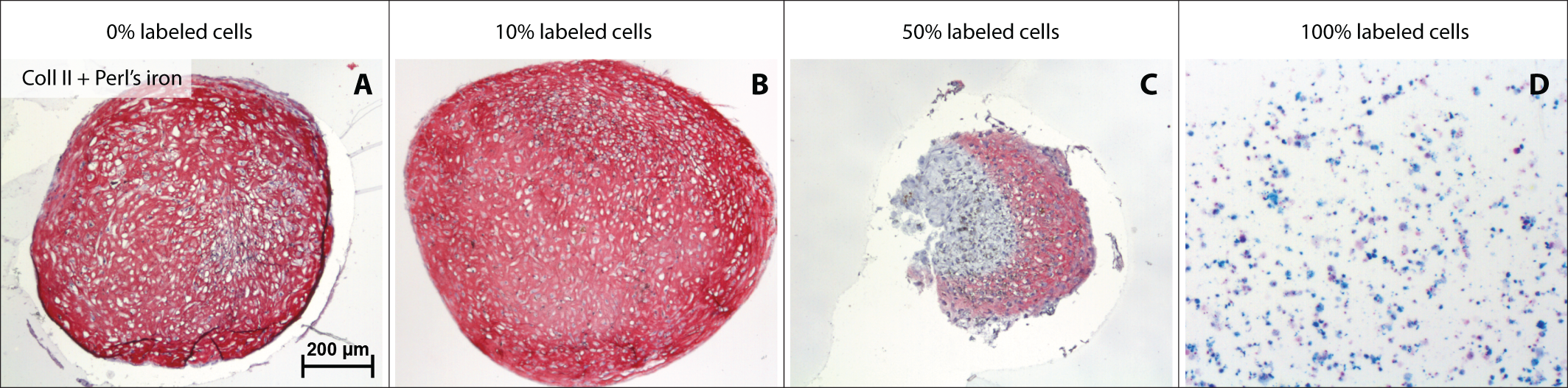

Supplement: Figure S3 — Chondrogenic differentiation of cryopreserved SPIO-labeled hBMSCs. Perl's iron stain (A–D) and collagen II immunohistochemistry (A–C) of chondrogenically differentiated cryopreserved hBMSCs. SPIO labeling of 10% of the cells did not influence pellet size or collagen II deposition compared to control cells (A and B). Using 50% of SPIO-labeled cells did negatively influence these outcome measures (C). Pellets consisting of 100% of labeled cells disintegrated within 7 days, showing viable and iron containing cells on a cytospin (D). Results shown for triplicate samples from two hBMSC donors. (TIF) [file pone.0017001.s003.tif]
